# Resident and migratory adipose immune cells control systemic metabolism and thermogenesis

**DOI:** 10.1038/s41423-021-00804-7

**Published:** 2021-11-26

**Authors:** Kevin Man, Axel Kallies, Ajithkumar Vasanthakumar

**Affiliations:** 1 Peter Doherty Institute for Infection and Immunity, Melbourne, VIC Australia; 2 Department of Microbiology and Immunology, University of Melbourne, Melbourne, VIC Australia; 3 Olivia Newton-John Cancer Research Institute, Melbourne, VIC Australia

**Keywords:** adipose tissue, immune cells, metabolism, Immunology, Inflammation

## Abstract

Glucose is a vital source of energy for all mammals. The balance between glucose uptake, metabolism and storage determines the energy status of an individual, and perturbations in this balance can lead to metabolic diseases. The maintenance of organismal glucose metabolism is a complex process that involves multiple tissues, including adipose tissue, which is an endocrine and energy storage organ that is critical for the regulation of systemic metabolism. Adipose tissue consists of an array of different cell types, including specialized adipocytes and stromal and endothelial cells. In addition, adipose tissue harbors a wide range of immune cells that play vital roles in adipose tissue homeostasis and function. These cells contribute to the regulation of systemic metabolism by modulating the inflammatory tone of adipose tissue, which is directly linked to insulin sensitivity and signaling. Furthermore, these cells affect the control of thermogenesis. While lean adipose tissue is rich in type 2 and anti-inflammatory cytokines such as IL-10, obesity tips the balance in favor of a proinflammatory milieu, leading to the development of insulin resistance and the dysregulation of systemic metabolism. Notably, anti-inflammatory immune cells, including regulatory T cells and innate lymphocytes, protect against insulin resistance and have the characteristics of tissue-resident cells, while proinflammatory immune cells are recruited from the circulation to obese adipose tissue. Here, we review the key findings that have shaped our understanding of how immune cells regulate adipose tissue homeostasis to control organismal metabolism.

## Introduction

While the primary function of the immune system is to protect against invading pathogens, several landmark studies have uncovered noncanonical functions of immune cells that extend beyond immune surveillance [1, 2]. Indeed, immune cells regulate several vital physiological processes, including tissue regeneration and repair, as well as organismal glucose metabolism [2, 3]. Adipose tissue is an endocrine and energy storage organ that is critical for the regulation of systemic metabolism. Impaired adipose tissue function is therefore closely linked to obesity and type 2 diabetes (T2D), both of which are both major public health problems in the developed and developing world. In addition, obesity is tightly linked to increased cancer incidence and impaired immune responses to infectious diseases [4, 5]. Lean adipose tissue secretes a variety of soluble mediators, including hormones, cytokines and chemokines, which regulate neuronal and metabolic circuits that control satiety, food intake, metabolite storage and catabolism [6, 7]. Notably, adipose tissue contains a diverse array of immune cells that directly impact its function. Thus, adipose tissue integrates organismal energy homeostasis with the immune system.

Adipose tissue can be found in many distinct anatomical locations and accordingly is heterogeneous in its composition and function. Visceral adipose tissue (VAT), for example, is located inside the abdominal cavity around the inner organs and plays a particularly important role in metabolism. Thus, impaired VAT function is tightly linked to metabolic disease [7, 8]. In contrast, subcutaneous adipose tissue (SCAT), which is found under the dermal layer of the skin, is particularly important for thermal regulation [9]. Indeed, all endothermic animals, including mammals and birds, use heat generated during cellular metabolism to maintain a stable core body temperature (homeothermy), which is critical for survival and allows for adaptation to diverse environmental climates [10, 11]. Most of the adipose tissue in adults consists of white adipose tissue (WAT), which is mainly an energy store. In contrast, brown adipose tissue (BAT), which is morphologically and transcriptionally distinct from WAT, has higher mitochondrial density and expression of mitochondrial uncoupling proteins with specialized functions in heat production (thermogenesis) [12]. Immune cells in adipose tissue also regulate thermogenesis by promoting beiging, which is a process in which WAT upregulates thermogenic transcriptional programs and mitochondrial uncoupling proteins to morphologically resemble BAT. Beige adipose tissue and BAT drive increased energy expenditure during cold exposure to maintain homeo-thermy, which is a major energy utilization program in endothermic mammals [12].

Adipose tissue is a multicellular organ composed of adipocytes, endothelial cells, mesenchymal stromal cells (MSCs) and immune cells [13]. Immune cells enriched in lean adipose tissue are largely anti-inflammatory and promote normal metabolic homeostasis. Many of these cells seed the tissue early in life and become permanently resident in adipose tissue [14]. During aging or the development of obesity, however, proinflammatory immune cells are progressively recruited to adipose tissue, which drives the development of insulin resistance [15]. These changes are in part driven by alterations in the secretome of adipose tissue, favoring the infiltration of immune cells that amplify adipose inflammation. For example, immune cell-derived proinflammatory cytokines such as TNF block insulin signaling by inactivating insulin receptor substrate (IRS), leading to insulin resistance and exacerbating blood glucose levels due to the downregulation of glucose transporters on adipocytes [16]. Thus, during chronic alterations in energy balance, the distribution and composition of adipose-resident immune cell populations is dramatically altered, which directly influences adipose tissue function. Here, we review the ongoing immunological characteristics of adipose tissue in healthy and obese contexts. Specifically, we discuss the differentiation and homeostatic requirements of immune cells and the multidirectional communication between immune cells, adipocytes, endothelial cells and stromal cells that regulate adipose tissue function and systemic metabolism. We propose a model in which adipose tissue homeostasis and function are regulated by an intricate and highly dynamic network of resident and recruited immune cells. Understanding the functional consequences of the interactions between immune and parenchymal cells in adipose tissue will further the development of new therapeutic strategies to treat obesity and diseases linked to obesity, including metabolic diseases and cancer.

## Tissue-resident immune cells preserve insulin sensitivity

WAT deposits, both visceral and subcutaneous, primarily function as sites of nutrient storage and lipid mobilization. These tissues also perform endocrine functions by secreting adipokines (leptin, adiponectin, resistin), lipokines (palmitoleic acid) and chemokines (CCL2) that are involved in modulating local and systemic immunity and metabolic homeostasis [2, 17–19]. While adipocytes themselves secrete many of these mediators, immune cells and MSCs produce multiple cytokines, such as interleukin-4 (IL-4), IL-5, TNF, IFNγ and IL-33, in response to the energy state of adipose tissue, which critically determines the metabolic fitness of organisms [20–23].

Myeloid cells are the most abundant adipose tissue-resident immune cells. Tissue-resident macrophages derive from the yolk sac and are the first immune cells to seed adipose tissue, where they undergo local expansion [24, 25]. In lean adipose tissue, macrophages display an alternatively activated M2 phenotype (CD206+CD301+CD11c−) and promote immune suppression. Under homeostatic conditions, M2 macrophages maintain adipocyte turnover by clearing dead adipocytes and debris through phagocytosis and lysosomal activation and by restraining the differentiation of adipocyte progenitors [26]. IL-4 produced by eosinophils preserves the alternative activation status of these macrophages, which in turn produce the anti-inflammatory cytokine IL-10 and the IL-1 decoy receptor to inhibit IL-1β signaling [22]. Adipocyte-derived adiponectin, which is abundant in lean adipose tissue, also polarizes macrophages to an M2 phenotype [27]. Several transcription factors are implicated in the differentiation of M2 macrophages. In addition to IL-4 and IL-13, the induced transcription factors STAT6, PPARγ, PPARδ, KLF4 and IRF4 are key drivers of M2 polarization [23, 28–31].

The role of conventional dendritic cells (cDCs) in adipose tissue homeostasis is still controversial [32]. This is partly due to difficulties in separating these cells from macrophages and monocytes. In most tissues, cDCs can be identified by high expression of CD11c and MHCII, but activated macrophages and monocytes in adipose tissue can also express prototypic DC markers [32, 33]. A recent study utilizing Zbtb46 reporter mice, in which cDCs can be distinguished from other myeloid cells with confidence, showed that both type 1 and type 2 conventional dendritic cells (cDC1s and cDC2s) play an anti-inflammatory role in lean adipose tissue, by producing IL-10 [34]. Notably, the anti-inflammatory function of cDC1s in adipose tissue requires the Wnt/β-catenin pathway (*Ctnnb1*) for IL-10 production, while cDC2s upregulate PPARγ to maintain a tolerogenic anti-inflammatory state in adipose tissue [34]. Thus, both tissue-resident macrophages and cDCs contribute to the maintenance of adipose tissue homeostasis.

Adipose tissue is also rich in innate lymphoid cells (ILCs), which are a major source of type 2 cytokines. Group 2 innate lymphoid cells (ILC2s) dominate lean adipose tissue and play an important role in preserving the T_H_2 milieu by regulating the recruitment of eosinophils, which in turn maintain M2 macrophages [35]. ILC2s depend on IL-33 and are important producers of the type 2 cytokines IL-5 and IL-13, which are thought to contribute to adipose tissue health [2, 35–37]. In addition, invariant natural killer T (NKT) cells, which produce IL-4, IL-13 and IL-10, are enriched in adipose tissue [38, 39]. Compared to splenic NKT cells, adipose NKT cells have a distinct phenotype and express the transcription factors Nfil3, T-bet and GATA3, while being negative for PLZF [39]. In a lean state, NKT cells preserve the M2 phenotype of macrophages by producing IL-10 in an Nfil3-dependent manner and facilitate the expansion of regulatory T cells by producing IL-2 [39]. Similar to those of NKT cells, lineage commitment and the differentiation of ILC2s also depend upon Nfil3, GATA3, T-bet and Id2, whereas PPARγ is critical for IL-33-dependent activation and functional licensing [40–42]. Notably, both ILC2s and NKT cells are bona fide adipose tissue-resident populations, as demonstrated by parabiosis experiments [36, 39].

Regulatory T (Treg) cells are the major anti-inflammatory adaptive immune cell subset enriched in lean adipose tissue. Tregs are specialized CD4+ T cells with suppressive and tissue-regulatory functions. In adipose tissue, these cells restrain inflammation to prevent the development of insulin resistance. Treg cells that reside in adipose tissue, in particular the VAT, display a unique phenotype as well as distinct transcriptional and cytokine requirements compared to their lymphoid tissue counterparts. These cells express high amounts of the adipocyte transcription factor PPARγ, which is essential for their development and maintenance [43, 44]. Indeed, the loss of PPARγ specifically in Tregs results in the specific loss of Tregs in VAT [44]. Unlike their lymphoid tissue counterparts, adipose Treg cells specifically require the cytokine IL-33 for survival and expansion [45]. The distribution, phenotype and homeostatic requirements of Treg cells in subcutaneous adipose tissue, however, are distinct from their counterparts in VAT [36]. The importance of Treg cells in controlling adipose inflammation and insulin resistance has been demonstrated by multiple studies. Systemic ablation of Treg cells using *Foxp3*^*DTR*^ mice or specific ablation within the adipose tissue using *Pparg*^*fl/fl*^*Foxp3*^*Cre*^ mice led to the development of insulin resistance during diet-induced obesity [43, 44]. We have shown that treatment of diet-induced or genetically obese mice with recombinant IL-33 could expand Treg cells in adipose tissue, restrain inflammation and revert glucose intolerance [46]. Expanding Treg cells systemically in obese mice using an IL-2 antibody complex also mitigated adipose inflammation and insulin resistance [43]. Consistent with the central role of Treg cells, pioglitazone, a PPARγ agonist that is used as an antidiabetic drug, was shown to exert its effects at least in part by activating PPARγ in adipose Treg cells [44]. Treg cells in adipose tissue express the enzyme hydroxyprostaglandin dehydrogenase (HPGD), which converts prostaglandin E_2_ (PGE_2_) into 15-keto PGE_2_, and Treg cell-specific loss of HPGD exacerbates adipose inflammation and insulin resistance in response to diet-induced obesity [47].

**Fig. 1:**
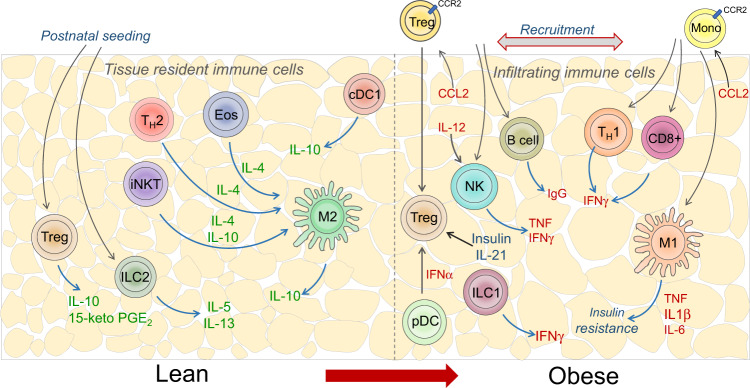
Immune spectrum in lean and obese adipose tissue. Lean adipose tissue is enriched in anti-inflammatory immune cells and immune cells that contribute to a T_H_2-dominant environment, including Treg cells, eosinophils, T_H_2 cells, M2 macrophages, ILC2s, NKT cells and γδ T cells, and are critical in preserving insulin sensitivity. Importantly, most of these immune cells migrate to adipose tissue early in life, where they establish tissue residency. Obesity displaces these immune cells by facilitating the recruitment of inflammatory immune cells such as M1 macrophages, T_H_1 cells, CD8+ T cells, NK cells, ILC1s and B cells. This effect is mediated at least in part by hypoxic and ER stress signals, as well as chemokines such as MCP-1/CCL2

Treg cells seed adipose tissue within the first weeks of life [45], increase in number during maturation, and decline during later stages of life [43, 48]. However, even in adult mice, Tregs are continuously recruited to adipose tissue [36]. Indeed, adipose Treg cells are known to arise from peripheral Treg cells that express low levels of PPARγ. After migrating to adipose tissue, these cells acquire the cardinal features of adipose Treg cells, including expression of the IL-33 receptor ST2 and the terminal differentiation marker KLRG1 [49, 50]. It is currently unclear whether Treg cells recruited during adulthood differ developmentally or functionally from Treg cells that seed adipose tissue during postnatal development. However, during all stages of development, T cell receptor (TCR) signaling is a vital requirement for adipose Treg cell differentiation and maintenance. This effect is mediated through the transcription factors BATF and IRF4, which are induced by TCR signaling, and activate PPARγ expression and IL-33 responsiveness by inducing the expression of ST2 [46]. Similarly, the transcription factor Blimp1, which is downstream of IRF4, preserves the transcriptional signature of Treg cells in adipose tissue by directly regulating the expression of ST2, PPARγ and IL-10 [36]. Recently, it was shown that insulin directly regulates the differentiation and function of adipose tissue Treg cells by inducing Hif1α-dependent PPARγ expression [51, 52]. Overall, lean adipose tissue is enriched in anti-inflammatory immune cells that are seeded early in life, display hallmarks of tissue residency and play a critical role in maintaining adipose tissue homeostasis and function (Fig. 1).

## Adipose tissue niches for immune cells

**Fig. 2:**
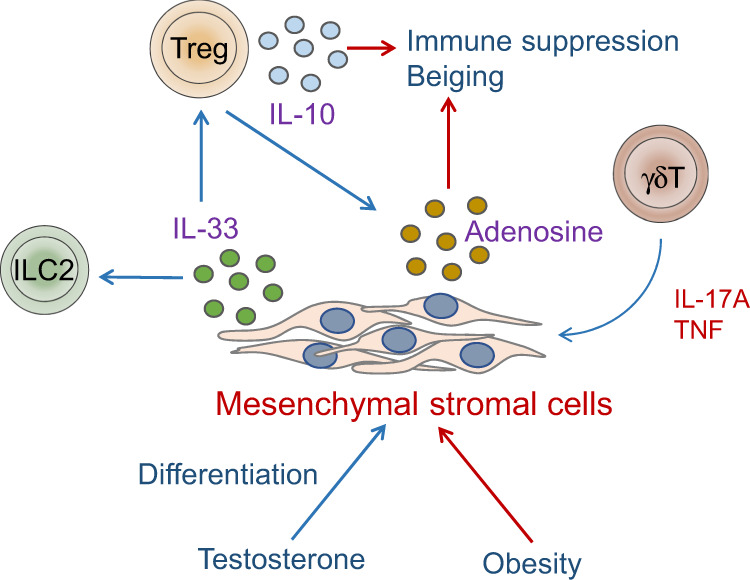
Immune-stromal cell crosstalk. PDGFRa+ mesenchymal stromal cells are the primary sources of IL-33 in adipose tissue. The cytokines TNF and IL-17A produced by γδT cells activate IL-33 production in MSCs. Treg cells and ILC2s rely on MSC-derived IL-33 for differentiation, maintenance, survival and activation. MSCs also express CD73, which catalyzes the production of adenosine, which coordinates with Treg cells to promote immune suppression in adipose tissue. Male sex hormones facilitate the differentiation of IL-33+CD73+MSCs, and obesity impairs this population

The notion that adipose tissue is enriched for an array of tissue-resident anti-inflammatory cell types suggests that this tissue contains specialized anatomical niches that promote the survival of these cells. One of the key mediators of such a niche may be IL-33, which plays a critical role in adipose tissue homeostasis by regulating the expansion and activity of ILC2s and Treg cells [2, 45, 46]. We and others have shown that PDGFRα+Pdpn+ MSCs are the main sources of IL-33 in adipose tissue [20, 36, 53, 54] (Fig. 2). Therefore, MSCs facilitate the accumulation of anti-inflammatory lymphocytes and directly contribute to sustaining the T_H_2 phenotype of immune cells and homeostasis in adipose tissue [36, 53]. Notably, we found that IL-33+ MSCs develop in a sex hormone-dependent manner, and the male sex hormone testosterone is critical for their differentiation [36]. Male mice show an enrichment in IL-33+ MSCs, and male but not female adipose tissue is specifically enriched in IL-33-dependent Treg cells [36]. On the other hand, ILC2s, which also rely on IL-33, did not show sexual dimorphism in their adipose distribution [36], indicating that other factors contribute to the sex-specific distribution and phenotype of Treg cells. Similarly, sex differences were not observed in Treg cells in SCAT, indicating unique sex hormone-mediated processes in VAT [36]. TNF and IL-17A production by PLZF-expressing γδ T cells was shown to be important for supporting IL-33 expression in MSCs [54] and therefore indirectly promoted immune suppression in adipose tissue (Fig. 2). Although this is just one example of the immune-stromal cell crosstalk that maintains tissue homeostasis and residency, it is likely that other factors contribute to the anti-inflammatory state and preserve the health and function of lean adipose tissue. Consistent with this idea, IL-33+ MSCs also exhibit high expression of the immunoregulatory molecule CD73 [36], an ectonucleotidase that converts AMP to adenosine [55], suggesting that MSCs play additional immunomodulatory roles distinct from IL-33-dependent regulation of immune cells (Fig. 2). CD73 is also highly expressed on a subset of VAT-resident Treg cells, thus contributing to adenosine production and beige fat biogenesis [52]. Interestingly, insulin signaling was shown to drive the transition of CD73^hi^ST2^lo^ to CD73^lo^ST2^hi^ Treg cells by inducing PPARγ expression [52], suggesting that VAT contains at least two subsets of Treg cells with distinct functions.

Notably, immune cells in adipose tissue niches have coopted aspects of the transcriptional machinery of adipocytes for their development and function. Indeed, key factors that are known to regulate adipogenesis and adipocyte function, including PPARγ and the Wnt signaling pathway [56], have been used by adipose-resident immune cells to promote various anti-inflammatory functions. These mechanisms may also confer immune cells with tissue tropic functions, which is evident from the critical role of PPARγ and IL-33 in adipose resident Treg cells. Thus, these adipose tissue-specific transcriptional networks, which are utilized by different cell types, may act as molecular links between adipocytes, MSCs and immune cells to enable tissue residency and communicate the physiological state of adipose tissue. For example, PPARγ is a molecule that controls lipid metabolism in adipocytes, MSCs and in immune cells [20, 28, 44]. Indeed, crosstalk with MSCs could be one of the mechanisms by which adipose immune cell residency and homeostasis are maintained.

## Adipose immune infiltration drives insulin resistance

Excess energy intake and low caloric output lead to obesity, a physiological condition marked by adipocyte hyperplasia (increased numbers) and hypertrophy (increased size). The growth and expansion of adipocytes results in hypoxia, the upregulation of oxidative and membrane/ER stress pathways and adipocyte death [57, 58]. Signals generated from stressed and dying adipocytes inhibit insulin action by initiating inhibitory serine phosphorylation of IRS proteins via JNK, MyD88 and IKK-β [59]. Furthermore, hypertrophic adipocytes upregulate chemokines, including CCL2, CCL5 and CCL8, leading to the recruitment of monocytes to adipose tissue, where these cells differentiate in situ into inflammatory M1 macrophages, which are a predominant source of TNF, IL-1β, IL-6 and IL-18 [24]. Inflammatory signals are then amplified locally and systemically, leading to the recruitment of other immune cells, including NK cells, ILC1s, B cells and CD8+ T cells [60–64], eventually replacing and inhibiting the function of resident M2 macrophages, Treg cells, NKT cells and ILC2s [2, 38, 43]. Obesity also results in dysregulated endocrine function. For example, obese adipose tissue exhibits reduced secretion of adiponectin, which mediates insulin sensitivity and is one of the most abundant adipokines secreted by adipocytes [65]. In contrast, the levels of the satiety hormone leptin are increased in obesity to counteract food intake [66, 67]. Notably, adipokines also have a direct impact on immune cell differentiation and activation. Leptin, for example, regulates multiple immune cells, including macrophages, NK cells and Treg cells [68]. Obesity therefore changes the local and systemic cytokine and adipokine environment and has profound implications on systemic metabolism, inflammation and immunity.

Macrophages are thought to be key mediators of adipose tissue inflammation and metabolic disease [69]. In obese adipose tissue, M1 macrophages are clustered around dead adipocytes and form crown-like structures, unlike resident M2 macrophages, which are interspersed between adipocytes and in the vasculature [70]. TNF production by macrophages recruited from the periphery is central to insulin resistance [21]. TNF inhibits glucose uptake by adipocytes by downregulating the expression of glucose transporters (slc2a4) [16] and reduce insulin signaling by inducing inhibitory serine phosphorylation of insulin receptor tyrosine kinase proteins [71]. Analogous to TNF, IL-6 can also inhibit insulin signaling by promoting serine phosphorylation of IRS proteins [72]. During obesity, recruited macrophages phagocytose adipocytes with high lipid levels to become lipid-laden macrophages [73]. Intracellular lipids are known to activate inflammatory pathways in adipose tissue macrophages [74], although precisely how M1 macrophages are activated has been a subject of controversy. Saturated fatty acid (SFA) signaling and TLR receptors play important roles in inflammatory gene activation in macrophages [75], while the activation of inflammasome (NLRP3)- and caspase-1-dependent pathways are important for mature IL-1β and IL-18 secretion by adipose macrophages [75]. Given the role of TLR4 in sensing not only LPS but also saturated fatty acids, it is widely believed that the SFA-TLR4 axis is involved in macrophage activation [76–78]. A recent paper, however, showed that SFAs do not activate macrophages via TLR4 but instead induce JNK signaling to reprogram macrophage metabolism during inflammation [79]. Accordingly, stress-induced JNK signaling is critical for the differentiation of M1 macrophages during obesity, and specific ablation of JNK in macrophages protected mice from diet-induced insulin resistance [80, 81].

While the M1 and M2 nomenclature provides a useful framework for the study of tissue macrophages, it is insufficient to describe the inflammatory status of adipose macrophages during obesity, as these cells often express markers of both M1 and M2 macrophages [14]. M1 macrophages are often identified by the expression of CD11c, and the ablation of CD11c+ cells (using CD11c-DTR mice) had a positive effect on ameliorating diet-induced insulin resistance [32]. However, CD11c is also expressed by conventional dendritic cells and monocytes, making it difficult to interpret some of these studies. While CD11c+ macrophages can be distinguished from dendritic cells by the expression of CD64 and MerTK, further investigations are required to delineate the functions of adipose macrophages and DCs. A recent study exploring macrophage heterogeneity using single-cell RNA-seq revealed a distinct inflammatory population that was CD9+Ly6C−, expressed genes related to lipid metabolism and was distributed in the obese adipose tissue of both mice and humans [82]. However, the precise function of this population has yet to be determined. Notably, inflammatory macrophages also contribute to the maintenance of adipose tissue homeostasis. During lipolysis and fasting-induced weight loss, inflammatory macrophages can phagocytose nonesterified fatty acids that are liberated during lipolysis and from dead adipocytes to prevent lipotoxicity [83]. Furthermore, blocking IL-6 trans-signaling prevented the accumulation of M1 macrophages but did not improve insulin tolerance [84]. Thus, the precise role of macrophages is likely to be multifaceted, and the signals that control their recruitment and function in adipose tissue remain a topic of great interest. Overall, further work is required to determine the developmental origin and function of this heterogeneous adipose myeloid population and to molecularly characterize distinct cell types that contribute to adipose tissue homeostasis and function.

Another important source of TNF during obesity, besides from macrophages, are NK cells, which are limited in distribution to epididymal VAT depots [85]. Obesity drives the upregulation of the NK cell activating receptor NCR1 on adipocytes. This, in turn, triggers IFNγ production by NK cells and facilitates the differentiation of inflammatory macrophages that promote insulin resistance [63]. Similarly, IFNγ produced by ILC1s reinforces inflammatory macrophage polarization [86] while simultaneously counteracting IL-33-mediated activation of ILC2s [37]. Accordingly, mice lacking *Nfil3,* which is critical for NK cell differentiation, or genetic loss of NCR1 or IFNγ resulted in improved insulin sensitivity [63], while treating mice with IL-15 to expand NK cells led to insulin resistance [85].

Although multiple lines of evidence suggest that macrophages and NK cells are critical in the initiation of adipose tissue inflammation and insulin resistance, many other cell types have been implicated in these processes. For example, B cells have been shown to expand in obese adipose tissue and promote the activation of M1 macrophages, as well as CD4+ and CD8+ T cells. B cell deficiency protected mice from the development of insulin resistance, whereas the transfer of pathogenic IgG from obese mice into B cell-deficient mice induced inflammation and insulin resistance [62]. Similarly, T cells play central roles in adipose tissue inflammation. For example, adipose infiltration of CD8+ effector T cells precedes macrophage infiltration, suggesting that CD8+ T cells initiate obesity-driven adipose inflammation [60]. Furthermore, CD4+ T cells, particularly T_H_1 cells, have been shown to play a proinflammatory role during obesity [87]. In line with this conclusion, deficiency of the transcription factor T-bet, which regulates the differentiation of T_H_1 cells and many other immune cell types [88], results in improved insulin sensitivity [87]. This effect appears to be intrinsic to CD4+ T cells, as adoptive transfer of wild-type CD4+ T cells promoted insulin resistance in *Rag*^*−/−*^ mice fed a high-fat diet, whereas the transfer of T-bet-deficient CD4+ T cells failed to initiate inflammation [87]. In support of a key role of T cells in metabolic disease, treating genetically obese *ob/ob* mice with anti-CD3 antibody minimized the expansion of T_H_1 cells and mitigated adipose inflammation and insulin resistance [61]. Collectively, multiple types of immune cells from the innate and adaptive arms of the immune system infiltrate adipose tissue during obesity to participate in the inflammatory cascade that culminates in insulin resistance and metabolic dysfunction (Fig. 1). In contrast, tissue-resident immune cell populations found in adipose tissue during homeostasis are critical for maintaining adipocyte homeostasis and function.

## Impaired immunosuppression mechanisms in obesity

Obesity-induced adipose inflammation and the influx of newly recruited inflammatory immune cells impair immunosuppressive mechanisms and further amplify inflammation and insulin resistance. Obesity has been shown to result in a loss of Treg cells and ILC2s specifically in VAT [2, 43]. Although the signals that lead to obesity-mediated Treg cell loss are not fully understood, excessive weight gain was found to drive the downregulation of *Pparg* and other adipose Treg cell-specific genes [48]. Obesity is also associated with the loss of IL-33-expressing MSCs, which affects not only Treg cells but also ILC2s [20, 36]. Finally, obesity results in increased IFNα production by plasmacytoid dendritic cells, which was shown to be toxic to Treg cells [89]. Hyperinsulinemia also directly impairs Treg cell population expansion and function, and accordingly, ablation of the insulin receptor specifically in Treg cells led to the expansion of adipose Treg cells, reduced adipose tissue inflammation and restored metabolic health [51]. Finally, IL-21 has been shown to be increased in adipose tissue during obesity, and IL-21 deficiency leads to the expansion of adipose tissue Treg cells and preserves insulin sensitivity [90]. Similar to Treg cells, ILC2s are impaired through the loss of IL-33-expressing MSCs during obesity [53]. Furthermore, IFNγ impairs the activation of ILC2s in adipose tissue during aging and obesity [37]. This, in turn, may also affect Treg cells, which are thought to interact with ILC2s through ICOS [37].

Interestingly, our recent work revealed an inflammation-driven pathway that recruits Treg cells to adipose tissue. Adipose tissue-derived CCL2 attracts Treg cells to adipose tissue [36], exploiting the same molecular pathway utilized by monocytes to infiltrate adipose tissue [36, 91]. Thus, the inflammation-mediated loss of Treg cells is counteracted by simultaneous de novo recruitment of peripheral Treg cells. Overall, however, Treg cell influx and expansion decline during the late stages of obesity and with physiological aging, allowing for the expansion of proinflammatory immune cells, exacerbating adipose tissue inflammation [43]. Importantly, the decline in adipose Treg cells and ILC2s during obesity is conserved across mice and humans [2, 43], suggesting that immune cell homeostasis is mediated by evolutionarily conserved mechanisms. In summary, obesity not only facilitates the infiltration of inflammatory immune cells into adipose tissue but also disables the protective mechanisms required to maintain insulin sensitivity and glucose homeostasis.

## Immune control of thermogenesis

In addition to energy storage and the regulation of systemic metabolism, adipose tissue also plays a critical role in thermogenesis, which is impacted by immune cells. Mammals have specialized heat-generating adipose tissue deposits, including brown and beige adipose tissue, which have high mitochondrial density and expression of mitochondrial uncoupling protein 1 (UCP1), a transmembrane protein that creates a proton channel in the mitochondrial inner membrane to allow the translocation of protons and the dissipation of the electrochemical gradient, leading to the uncoupling of oxidative phosphorylation from the synthesis of ATP and the generation of heat as a byproduct [92]. Upon environmental cold exposure or activation of the sympathetic nervous system via beta-3 adrenergic stimulation, inguinal or subcutaneous WAT deposits can also engage in adaptive thermogenesis by upregulating the expression of UCP1. UCP1^+^ cells are known as ‘beige’ adipocytes and are transcriptionally distinct from white or brown adipocytes [93, 94]. These cells are derived from Myf5^−^ PDGFRα^+^ precursor cells [95, 96] or by the direct conversion or transdifferentiation of existing white adipocytes [97, 98]. However, despite its central role in thermogenesis, UCP1 is not essential because *Ucp1*^*−/−*^ mice show no defects in adaptation to long-term cold exposure [99]. UCP1-independent mechanisms of thermogenesis occur predominantly in the form of futile metabolic cycling processes, during which tandem inverse reactions occur simultaneously, and the only net effect is the hydrolysis of ATP and dissipation of energy in the form of heat [100–103].

**Fig. 3:**
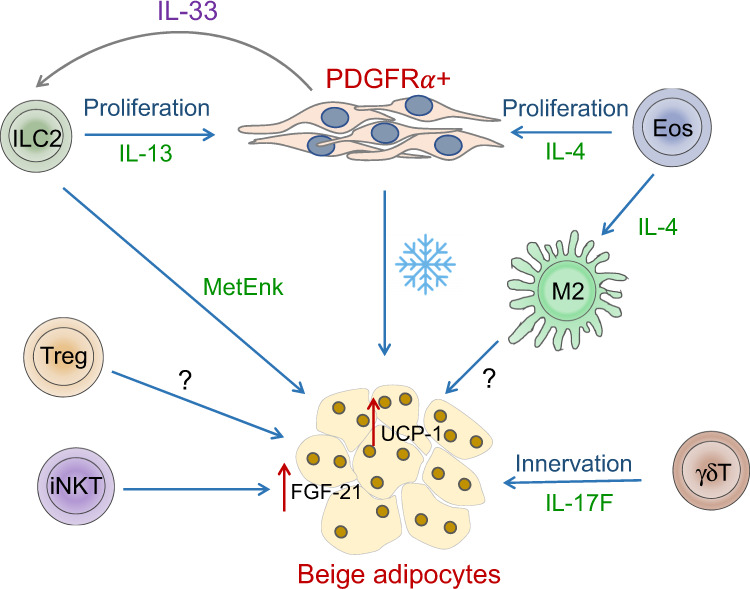
Immune regulation of thermogenesis. Upon cold exposure, M2 macrophages promote the differentiation of beige adipocytes from PDGFRα+ stromal cells. This process is mediated by IL-4 produced by eosinophils. IL-4 and IL-13 derived from eosinophils and ILC2s also promote the proliferation of PDGFRα+ cells to facilitate beiging. While the mechanism by which M2 macrophages promote beiging is unclear, ILC2s and NKT cells promote the upregulation of UCP-1 and FGF-21 in adipose tissue, respectively. Innervation mediated by γδT cell-derived IL-17F also promotes beiging and thermogenesis. The role of Treg cells in thermogenesis is controversial

In a lean state, adipose tissue-resident immune cells participate in the regulation of adaptative thermogenesis predominantly via the secretion of cytokines that influence the differentiation and function of mesenchymal stromal cells and adipocyte precursor cells or by controlling the differentiation and phenotype of other adipose-resident immune cells, indirectly impacting adipocyte precursors. M2 macrophages support the terminal differentiation of PDGFR*α*^+^ stromal cells to beige adipocytes upon cold exposure [104, 105] (Fig. 3). In a related circuit, eosinophil-derived IL-4, together with ILC2-derived IL-13, stimulates the proliferation and differentiation of PDGFR*α*+ stromal cells to the beige adipocyte lineage [104, 106]. Accordingly, mice lacking eosinophils, IL-4 and IL-13, or IL-4Rα, or mice with a macrophage-specific deletion of IL-4r*α,* exhibit deficiencies in beige adipocyte formation, cold-induced thermogenesis and decreased energy expenditure [106] (Fig. 3). IL-33-dependent ILC2s are also necessary for sustaining the proliferation and commitment of PDGFR*α*+ adipocyte precursor cells to the beige lineage [106]. Additionally, IL-33 induces the expression of methionine-enkephalin peptides in ILC2s, which induces beige fat biogenesis via an unknown mechanism [107]. Recently, *γδ*TCR T cells were shown to be important for regulating thermogenesis by directing the innervation of BAT and by increasing the expression of tyrosine hydroxylase through IL-17F- and adipocyte IL-17Rc-dependent signaling [108] (Fig. 3). Finally, activated NKT cells contribute to WAT beiging by increasing the expression of FGF21, a hormone involved in stimulating adipocyte glucose uptake [109] (Fig. 3).

Adipokines produced by adipocytes can also induce thermogenesis and increase energy expenditure. For example, adiponectin is increased in subcutaneous WAT by chronic cold exposure and promotes beige adipocyte formation and BAT UCP1 expression via its effects on macrophage polarization, which increases M2 macrophage polarization in WAT without affecting type 2 cytokine expression or ILC2 abundance [110]. BAT also contains resident innate and adaptive immune cells, although their tissue tropic functions are not well understood. For example, BAT harbours transcriptionally distinct Treg cells that regulate the inflammatory tone of the tissue, although Treg depletion seems to have only minor effects on BAT thermogenic gene programs and thermogenesis [111, 112]. It remains to be determined whether BAT-resident macrophages and ILC2s also participate in regulating thermogenesis in a similar manner to their counterparts in WAT.

Energy balance is a critical regulator of the extent of adaptive thermogenesis in adipose tissue. Inflammatory pathways activated by obesity inhibit adaptative thermogenic programs. For example, IL-1*β* and TNF signaling in adipocytes inhibits UCP-1 expression and cold-induced thermogenesis [113, 114]. In a similar manner, IκB kinase-*ɛ* (IKK*ɛ*)-, IRF3- and TGF-*β*-dependent inflammatory pathways inhibit adipose tissue thermogenesis and energy expenditure during obesity, and blocking these pathways stimulates adipose tissue thermogenesis [115, 116]. Interestingly, exercise and energy restriction regimens (caloric restriction or intermittent fasting) promote beige adipocyte formation and energy expenditure through immune cell-derived cytokines. Specifically, the positive effect of exercise and fasting on thermogenesis requires IL-4 production by eosinophils and alternative activation of macrophages in WAT [104] and signaling in an IL-4R*α*- and STAT6-dependent manner [105]. Exercise was also shown to increase the production of meteorin-like hormone in muscle and adipose tissue, which stimulated the differentiation of M2 macrophages, thereby indirectly increasing beige differentiation and thermogenic capacity [105]. Intermittent fasting, while acting on the IL-4/IL-13 signaling cascade, also influences microbiota composition and enhances hepatic production of FGF21 to increase beiging in a PPAR*α*-dependent manner [117]. Whether exercise and caloric restriction regimens directly influence adipocyte thermogenesis and the mechanisms remain to be elucidated.

While fasting and caloric restriction are associated with increases in health span and longevity [118], physiological aging induces a decline in the thermogenic capacity of adipose tissue, which is accompanied by increased accumulation of inflammatory B cells, αβ-TCR T cells and M1 macrophages with senescence-associated gene signatures and pathways associated with catecholamine catabolism to suppress lipolysis [119–121]. With the increase in inflammatory senescent immune cells in adipose tissue, there is an accompanying decline in M2 macrophages and ILC2s, which are important for preserving thermogenic capacity, as detailed above. ILC2s become dysfunctional during aging, and accordingly, IL-33-mediated expansion of aged ILC2s failed to promote thermogenesis in aged mice. Accordingly, the transfer of ILC2s from young mice restored thermogenic capacity in aged mice during cold exposure [122].

Similar to insulin signaling, the thermogenic capacity of adipose tissue also depends upon the activity of various anti-inflammatory resident immune cells, which are impaired by inflammatory signaling cascades that are upregulated during obesity and decline in function with physiological aging. Overall, adipose tissue immune cells have indispensable roles in regulating beige and brown thermogenic adipocyte differentiation, thermogenic capacity and systemic energy expenditure by regulating the sympathetic innervation of adipose tissue and the differentiation of thermogenic PDGFR*α*+ adipocyte precursor cells.

## Controversies and open questions

Adipose tissue contains a multitude of different immune and stromal cells and is impacted by factors such as diet, age and sex. Most studies, however, focus on the roles of only one or a few different cell types under one set of conditions. Thus, it is not surprising that controversies have arisen about the relative impacts of certain cell types on adipose tissue functions. Conflicting results are most likely due to differences in experimental design. Sex in particular plays a critical role, and male mice are far more susceptible to the development of insulin resistance and obesity than female mice [36, 123]. This difference has been attributed to adipose tissue-intrinsic differences [124] and differences in the abundance and phenotype of Tregs and stromal cells in VAT [36]. Another important factor is age. NKT cells are particularly abundant in adipose tissue in mice between 8 and 16 weeks of age [43], while Treg cells accumulate until 6–8 months of age before declining [43]. Similarly, the length of dietary interventions or the microbiota [125], the use of nonlittermate controls or differences in the experimental readouts [126] have been shown to play critical roles in the outcomes of studies of adipose tissue function.

Even understanding the role of individual cell populations is not without challenges. NKT cells, for example, can exert both pro- and anti-inflammatory effects, and given their abundance in lean VAT in mice and humans [38, 127], several studies have examined their role in obesity-induced inflammation with partially contradictory results [125, 128–133]. This discrepancy may be due to differences in the genetic models used to deplete NKT cells (*Cd1d*^−/−^ or *Jα18*^*−/−*^ mice), which lack different types of NKT cells [134]. Critically, the ablation of CD1d specifically on adipocytes resulted in inflammation and insulin resistance, indicating an adipocyte-intrinsic role for CD1d [135]. Single-cell sequencing technologies to understand the heterogeneity of different NKT cell subsets and the use of cell type-specific knockout models are important to specifically target NKT cells during early and established obesity to fully understand their role in metabolic regulation.

Similar to NKT cells, the role of Treg cells is also somewhat controversial in the context of adipose tissue biology. Although these cells are widely accepted as mediators that suppress inflammation, including that in adipose tissue [136], they have also been implicated in exacerbating adipose inflammation [137, 138]. The examination of mice with Treg cell-specific ablation of PPARγ suggested a negative role of adipose tissue Treg cells during aging [137]. Similarly, two studies suggested that Treg cells dampen adipose tissue beiging in a Blimp1- and IL-10-dependent manner [137, 139]. In our own experiments, the loss of Blimp1 in Treg cells resulted in the depletion of adipose tissue-resident Treg cells and led to impaired systemic glucose homeostasis [36]. One must consider alternative explanations for the observed phenotypes of mice with Treg cell-specific deletion of Blimp1 or IL-10. Deletion of both molecules is known to affect Treg cells in many tissues, including in the gastrointestinal tract [140], thus resulting in tissue inflammation and weight loss and indirectly contributing to increased glucose metabolism. Similarly, it is possible that Treg cells that are impaired by aging or the loss of critical regulatory molecules, such as PPARγ, lose expression of their lineage-defining transcription factor Foxp3 and acquire an inflammatory phenotype, subsequently contributing to adipose inflammation. Fate mapping mouse models would help in understanding the abundance of ex-Treg cells and their contribution to inflammatory status in different mouse models. Finally, it is currently unclear whether regulatory circuits that are active in murine Treg cells also contribute to Treg cell differentiation and function in human adipose tissue. For example, our data suggested that IL-33 signaling plays a role in mouse and human adipose tissue Treg cells [46], while others have failed to identify ST2+ Treg cells in human adipose tissue [141]. Given the paucity of Treg cells in female and obese adipose tissue [36, 43, 46], future studies need to take into account the sex and adiposity of human subjects. In-depth characterization of immune cells embedded in human adipose tissue will help to further the understanding of evolutionarily conserved immune-mediated mechanisms. Single-cell genomic technologies may further illuminate the complexity of immune cells in human adipose tissue and variations associated with sex, age and adiposity. Finally, the role of Treg cells in thermogenesis is controversial and requires further investigation. While one study has shown that the loss of Treg cell function (Blimp1 or IL10 deletion) improves thermogenesis [138], another study demonstrated that adoptive transfer of Treg cells promoted the beiging of subcutaneous adipose tissue [142].

There is a common idea that lean adipose tissue is T_H_2-biased, and obesity promotes T_H_1-mediated inflammation. Indeed, several types of immune cells in lean adipose tissue produce T_H_2 cytokines, such as IL-4, IL-13, IL-5 and IL-10 [2, 38, 44]. MSCs also contribute to T_H_2 inflammation in lean adipose tissue by producing IL-33 [20, 36, 53]. Consistent with the T_H_2 profile, immune cells such as ILC2s and Treg cells also express the T_H_2 transcription factor Gata3 in lean adipose tissue [2, 36]. While systemic loss of T-bet protected against the development of insulin resistance [87], the ablation of IFNγ, the major T_H_1 cytokine, only modestly improved insulin sensitivity in obese mice [143]. However, there have been no systematic studies that have examined the role of T_H_1 and T_H_2 cytokines in female mice. Given the pronounced differences in susceptibility to metabolic diseases [36, 144] and in VAT Treg cell phenotype and function between males and females [36], revisiting the T_H_1/T_H_2 model in adipose tissue health and disease is urgently needed.

Notably, adaptive thermogenesis influences immune recruitment and composition in adipose and peripheral tissues, including the liver and gastrointestinal tract [145–147]. Modulating thermogenic programs, such as by increasing housing temperature to the thermoneutral zone, can alter phenotypes driven by obesity-induced inflammation, atherosclerosis, bacterial sepsis, nonalcoholic fatty liver disease, colitis and cancer [145, 147, 148] to those more consistently observed in human physiology. Normal vivarium conditions impose significant thermal stress on experimental animals, which can obscure experimental results and represent an additional obstacle for predictive modeling of human diseases and therapies, as humans spend most of their lives under thermoneutral conditions [149]. Thermogenesis and ambient housing temperature, therefore, should be experimental variables that are carefully considered when carrying out metabolic and immunological studies.

Finally, it must be acknowledged that there are certain limitations in regard to animal models and their use in obesity and metabolic studies. For example, there are differences in pancreatic islet architecture in mice compared with humans [150]. Furthermore, genome-wide association studies showed that human obesity is polygenic in nature and associated with over 100 candidate genes, making monogenetic mouse models of obesity less amenable to therapeutic translation [151]. Therefore, the validation of findings based on murine adipose tissue is required in both healthy and obese humans of both sexes to fully understand the role of the adipose immune system in regulating metabolism and obesity-related chronic inflammatory diseases. Overall, many questions remain, and further work is required to untangle the complicated relationships between adipose tissue function and the immune system.

## Concluding remarks

Over the last two decades, several landmark studies have uncovered the remarkable impact of the immune system on systemic metabolism, as outlined in this review. The quest to understand the precise role of each immune cell subset in protecting adipose tissue homeostasis or contributing to adipose inflammation and associated pathology has revealed cellular networks involving immune cells, adipocytes and MSCs. It has become apparent that most immune cells that are anti-inflammatory and contribute to the preservation of systemic glucose metabolism populate adipose tissue early in life, expand locally and are not frequently replenished by circulating immune cells. The adaptation to adipose tissue microenvironments and tissue-derived signals, as well as the use of molecular regulators, such as IL-33 and PPARγ, are common in many of these adipose tissue-resident immune cells. Stromal cells may play an important yet poorly defined role in mediating the development, maintenance, and intercellular communication of adipose tissue immune cells.

Reestablishing immunoregulatory mechanisms in adipose tissue could be a therapeutic approach to treat metabolic inflammation and insulin resistance. Additionally, there is great interest in understanding the cellular and molecular pathways that increase and sustain beige and brown adipocyte thermogenesis as a mechanism to regulate energy expenditure and metabolism in humans, and there are potential implications for the management of obesity and diabetes [152]. Overall, understanding the population dynamics of immune cells and their functions in adipose tissue will aid in the design of novel therapeutic interventions that dampen adipose inflammation to restore insulin sensitivity and glucose homeostasis.
