# Correction: Resident and migratory adipose immune cells control systemic metabolism and thermogenesis

**DOI:** 10.1038/s41423-022-00844-7

**Published:** 2022-02-25

**Authors:** Kevin Man, Axel Kallies, Ajithkumar Vasanthakumar

**Affiliations:** 1 Peter Doherty Institute for Infection and Immunity, Melbourne, VIC Australia; 2 Department of Microbiology and Immunology, University of Melbourne, Melbourne, VIC Australia; 3 Olivia Newton-John Cancer Research Institute, Melbourne, VIC Australia

**Keywords:** Immunology, Inflammation

Correction to: *Cellular & Molecular Immunology* 10.1038/s41423-021-00804-7, published online 26 November 2021

The article Resident and migratory adipose immune cells control systemic metabolism and thermogenesis, written by Kevin Man, Axel Kallies and Ajithkumar Vasanthakumar was originally published electronically on the publisher’s internet portal on 24.11.2021 without open access. With the author(s)’ decision to opt for Open Choice the copyright of the article changed on to © The Author(s) 2021 and the article is forthwith distributed under a Creative Commons Attribution.

Open Access This article is licensed under a Creative Commons Attribution 4.0 International License, which permits use, sharing, adaptation, distribution and reproduction in any medium or format, as long as you give appropriate credit to the original author(s) and the source, provide a link to the Creative Commons licence, and indicate if changes were made.

The images or other third-party material in this article are included in the article’s Creative Commons licence, unless indicated otherwise in a credit line to the material. If material is not included in the article’s Creative Commons licence and your intended use is not permitted by statutory regulation or exceeds the permitted use, you will need to obtain permission directly from the copyright holder.

